# The Structure and Evolution of Buyer-Supplier Networks

**DOI:** 10.1371/journal.pone.0100712

**Published:** 2014-07-07

**Authors:** Takayuki Mizuno, Wataru Souma, Tsutomu Watanabe

**Affiliations:** 1 National Institute of Informatics, Tokyo, Japan; 2 Department of Informatics, The Graduate University for Advanced Studies, Tokyo, Japan; 3 PRESTO, Japan Science and Technology Agency, Tokyo, Japan; 4 College of Science and Technology, Nihon University, Chiba, Japan; 5 Graduate School of Economics, University of Tokyo, Tokyo, Japan; 6 The Canon Institute for Global Studies, Tokyo, Japan; University of Warwick, United Kingdom

## Abstract

In this paper, we investigate the structure and evolution of customer-supplier networks in Japan using a unique dataset that contains information on customer and supplier linkages for more than 500,000 incorporated non-financial firms for the five years from 2008 to 2012. We find, first, that the number of customer links is unequal across firms; the customer link distribution has a power-law tail with an exponent of unity (i.e., it follows Zipf's law). We interpret this as implying that competition among firms to acquire new customers yields winners with a large number of customers, as well as losers with fewer customers. We also show that the shortest path length for any pair of firms is, on average, 4.3 links. Second, we find that link switching is relatively rare. Our estimates indicate that the survival rate per year for customer links is 92 percent and for supplier links 93 percent. Third and finally, we find that firm growth rates tend to be more highly correlated the closer two firms are to each other in a customer-supplier network (i.e., the smaller is the shortest path length for the two firms). This suggests that a non-negligible portion of fluctuations in firm growth stems from the propagation of microeconomic shocks – shocks affecting only a particular firm – through customer-supplier chains.

## Introduction

Firms in a modern economy tend to be closely interconnected, particularly in the manufacturing sector. Firms typically rely on the delivery of materials or intermediate products from their suppliers to produce their own products, which in turn are delivered to other downstream firms. Two recent episodes vividly illustrate just how closely firms are interconnected. The first is the recent earthquake in Japan. The earthquake and tsunami hit the Tohoku region, the north-eastern part of Japan, on March 11, 2011, resulting in significant human and physical damage to that region. However, the economic damage was not restricted to that region and spread in an unanticipated manner to other parts of Japan through the disruption of supply chains. For example, vehicle production by Japanese automakers, which are located far away from the affected areas, was stopped or slowed down due to a shortage of auto parts supplies from firms located in the affected areas. The shock even spread across borders, leading to a substantial decline in North American vehicle production. U.S. Federal Reserve Chairman Ben Bernanke, for example, stated in the aftermath of the earthquake: “U.S. economic growth so far this year looks to have been somewhat slower than expected. Aggregate output increased at only 1.8 percent at an annual rate in the first quarter, and supply chain disruptions associated with the earthquake and tsunami in Japan are hampering economic activity this quarter.” (Speech at the International Monetary Conference, Atlanta, Georgia, on June 7, 2011). The second episode is the recent financial turmoil triggered by the subprime mortgage crisis in the United States. The adverse shock originally stemming from the so-called toxic assets on the balance sheets of U.S. financial institutions led to the failure of these institutions and was transmitted beyond entities that had direct business with the collapsed financial institutions to those that seemed to have no relationship with them, resulting in a storm that affected financial institutions around the world.

The lesson from these two episodes is that national economies, as well as the global economy, are subject to the risk of a chain-reaction in product disruptions through customer-supplier linkages. Such risk is especially high when the linkage structure in the economy is dominated by a few hub firms whose products are supplied to many other firms as input. Importantly, supply chain disruptions are more serious when there are no close substitutes to these hub firms, at least in the short run. Motivated at least partly by the two episodes, some recent studies in economics have sought to develop theoretical models on production chains that extend input-output analysis, which dates back to the seminal work by Wassily Leontief published in the 1930s [1], to identify conditions under which microeconomic shocks, i.e., idiosyncratic shocks to individual firms, can propagate to the rest of the economy through production chains, leading to fluctuations in production at the macro level [2]–[7]. Policymakers have also started to think about how to prepare for the propagation of adverse shocks through production chains. The study of networks as phenomena that deserve analysis goes back to the small-world network model by Watts [8] and has gained popularity in a variety of scientific disciplines, including statistical physics, computer science, biology, and sociology. The methodology developed in those disciplines has been introduced into economics only relatively recently [9], [10], but has produced important contributions to the literature on bank-firm relationships [11], on cross shareholdings [12], on supply chains [13]–[17], on systemic risks in financial markets [18], [19], and on international trade [20]–[22].

**Figure 1:**
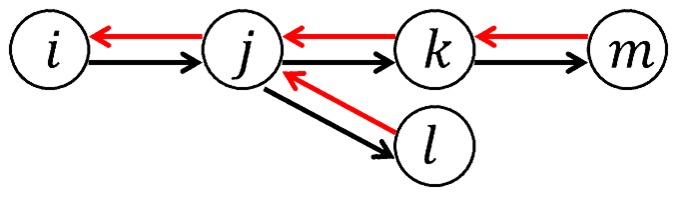
An illustration of customer-supplier network consisting of five firms. The red arrows in the figure indicate the flow of money, while the black arrows indicate the flow of products each firm produces. For example, firm 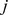 purchased something from firm 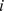 and sells something to firms 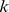 and 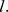

Against this background, the present study seeks to provide empirical evidence on the structure and evolution of customer-supplier networks in Japan using a unique dataset that contains information on customer and supplier linkages for more than 500,000 incorporated non-financial firms for the five years from 2008 to 2012. This dataset provides the customer and supplier lists for each firm. We use these lists to produce a customer-supplier network. To illustrate this, Figure 1 shows a simple example consisting of five firms, which are represented by the circles. The red arrows indicate the flow of money, while the black arrows indicate the flow of products each firm produces. Firm 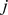 purchases something from firm 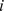 and sells something to firms 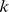 and 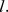 That is, for firm 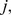 firm 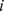 is a supplier and firms 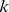 and 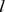 are both customers. Moreover, firm 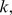 which purchases something from firm 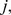 sells to firm 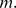 Note that, in this simple example, firm 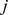 is not only a customer but also a supplier, and the same thing is true for firm 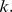 In the actual data, most of the firms are a customer and a supplier simultaneously through directed links between firms, which will be shown later. The rest of the paper is organized as follows. Next section provides a more detailed description of the dataset, and looks at the basic structure of customer-supplier networks, paying particular attention to how closely firms are interconnected. We move on to the issue of how customer-supplier networks evolve over time. We empirically evaluate to what extent firm sales and growth are affected by the propagation of idiosyncratic shocks through production chains. The last section concludes the paper.

## Analysis

### Data

The dataset we use is jointly compiled by Teikoku Databank, Ltd. (TDB), one of the largest business database companies in Japan, and the HIT-TDB project of Hitotsubashi University. The dataset mainly provides information related to corporate bankruptcies and credit ratings and covers about 1.3 million incorporated non-financial firms. Since the number of corporations in Japan in 2006 (as reported in the 2006 Establishment and Enterprise Census) was 1.493 million, our dataset covers about 90 percent of all incorporated firms in Japan. TDB collects various kinds of information from these firms, including annual or more frequent financial statement data.

Two types of information on customer-supplier relationships are recorded in this dataset. First, the dataset contains information on the number of three types of relationships a firm has with other firms, namely relationships with customers (i.e., firms to which a firm sells its products), suppliers (i.e., firms from which a firm purchases raw materials and intermediate products), and owners (i.e., firms by which a firm is owned). Since in this paper we focus on customer-supplier relationships, we mainly use information on customer and supplier linkages. We denote the total number of firm *i*'s customer links by 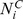 and the total number of supplier links by 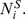 Second, the dataset lists the firms with which a firm has links (i.e., customers or suppliers to the firm) with their identification codes. However, the list is not exhaustive and its length cannot exceed 60 firms. This means that for smaller firms with fewer than 60 partners all of their partners are listed, but for large firms with more partners only the 60 most important ones are listed. In all cases, transaction partners are listed in descending order of importance based on the transaction volume.

Table 1 presents descriptive statistics on customer and supplier linkages. All statistics in the table are calculated using the total number of linkages, that is, 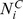 and 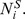 Note that the table provides linkage information for five different years (i.e., 2008, 2009, 2010, 2011, and 2012), allowing us to investigate not only the structure of customer-supplier networks at a particular point in time but also their evolution. The sample mean for the number of customer links per firm is about 340 each year, and the median for the number of customer links per firm is 50, which is about one seventh of the mean, implying that the customer link distribution is not symmetric, but is substantially skewed to the right. In fact, the maximum number of customer links in 2012 was 95,512, which is far greater than the mean or the median, given that the standard deviation is only 2,053. Turning to the number of supplier links, the sample mean is about 60 each year, which is much smaller than the number of customer links. A typical firm has six times as many customer links as supplier links. The median number of supplier links per firm is 20, implying again that the distribution for the number of supplier links is not symmetric but is skewed to the right. The maximum number of supplier links per firm is also much greater than the mean or the median.

**Table 1:** Number of customer and supplier links per firm.

Customer Links					
	2008	2009	2010	2011	2012
Number of firms	160,508	155,806	144,006	142,931	145,317
Number of links per firm					
Mean	339	343	341	340	339
Median	50	50	50	50	50
Std. Dev.	2,107	2,090	2,015	2,022	2,053
Max.	90,200	90,504	90,000	90,000	95,512
Min.	0	0	0	0	0

Supplier Links					
	2008	2009	2010	2011	2012
Number of firms	215,562	208,459	192,111	189,493	193,045
Number of links per firm					
Mean	56	58	61	62	61
Median	20	20	20	20	20
Std. Dev.	281	314	368	332	351
Max.	52,100	55,100	70,000	70,000	70,000
Min.	0	0	0	0	0

To investigate the structure of customer-supplier networks and their evolution over time, we use the list of firms linked to a firm with their identification codes. As mentioned, the list is not exhaustive, so that, as far as large firms are concerned, links with less important partners are not recorded. The number of customers and suppliers in the list is 6.7 and 6.4 for a typical firm, which is much smaller than the means of the *total* number of customer and supplier links presented in Table 1. We augment the customer/supplier lists as follows. We first identify firm A as a supplier of firm B using the *customer* list of firm A, thereby producing an augmented supplier list of firm B. We add up the number of customer links originally shown in the customer list of a firm and the number of customer links identified in this way, and denote the sum by 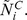 Similarly, we use the supplier lists of firms to produce augmented customer lists and define 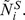 This kind of “reverse lookup” method has been applied to different datasets in previous studies on interfirm relationships, including [14]–[16]. Comparing 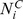 and 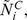 we observe a relationship of the following form:
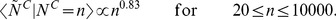
(1)where 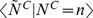 represents the mean of 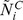 across 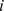 given that the total (true) number of customer links, 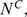 for those firms is equal to 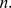 Interestingly, the power exponent of 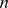 is smaller than unity, implying that for firms with a large number of customers the augmented list still does not capture the true number of customers. The example of a firm leasing vending machines to other firms explains why. This firm has a very large number of customer firms, but because vending machines are not regarded as a key input to production by most customer firms, they do not include the leasing firm in their list of suppliers. In this case, 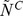 for the leasing firm is much smaller than 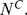


Turning to supplier lists, we have
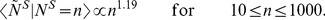
(2)indicating that the exponent of 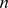 is now greater than unity, which means that 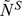 more than doubles when 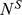 doubles, and in this sense 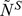 overestimates 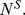 A likely reason is that small suppliers to a prestigious firm with a large number of suppliers will include the prestigious firm in their customer list reported to TDB, since the prestigious firm is regarded as a key constituent of their customer base. However, this effect will be weak or absent if a customer firm is not that prestigious, which makes the exponent of 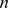 in Eq. (2) greater than unity.

### The Structure of Customer-Supplier Networks

#### Unequal links across firms

**Figure 2:**
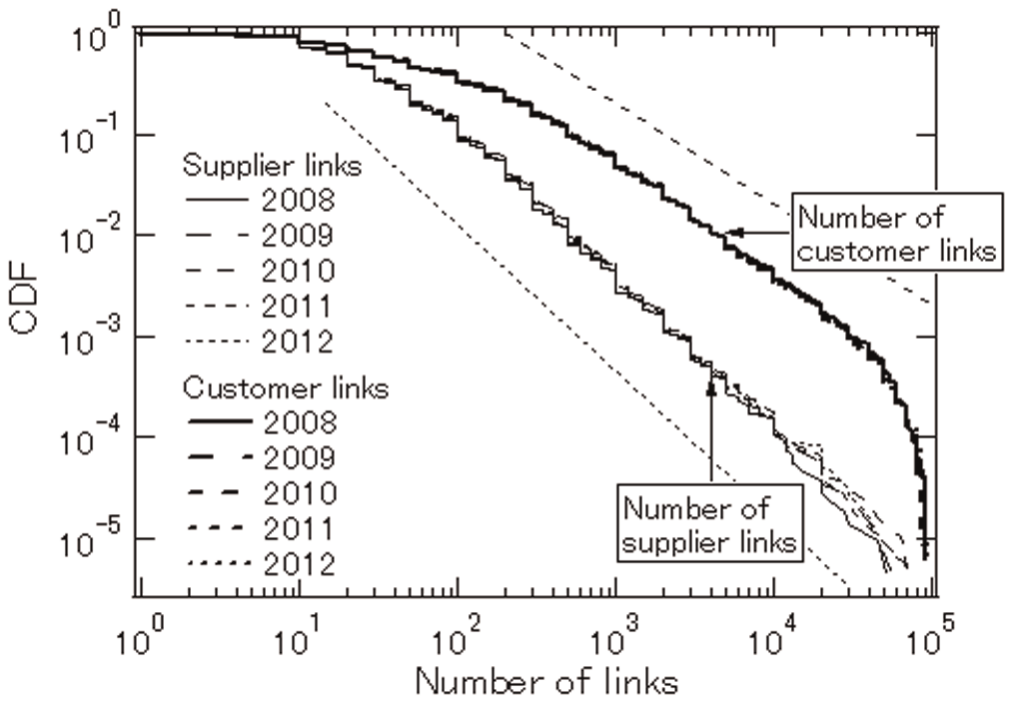
Cumulative distributions of customer and supplier links in 2008–2012. The horizontal axis represents the *total* number of links, i.e., 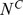 and 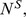 while the vertical axis represents the corresponding cumulative densities. The dotted straight lines are reference lines with a slope of −1 and −1.5 respectively. The number of firms used in this calculation is shown in Table 1.

The number of links is unequal across firms with regard to both customer and supplier linkages, as we saw in Table 1. One may wonder how unequal it is across firms and whether the degree of inequality differs between customer and supplier linkages. To address these questions, we show in Figure 2 the cumulative distribution functions (CDFs) of links across firms. The horizontal axis represents the number of links, while the vertical axis shows the corresponding cumulative densities. The horizontal and vertical axes are both in logarithm. For example, the number on the vertical axis corresponding to 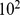 on the horizontal axis is about 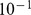 for supplier linkages, indicating that firms with more than 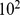 supplier links account for one tenth of all firms. The figure shows the CDFs for the customer and supplier linkages for each of our five observation years (2008, 2009, 2010, 2011, and 2012).

Given that the mean for the logarithm of the number of customer links is 1.72 and the corresponding standard deviation is 0.783, a number like 5,000 links deviates from the mean by more than 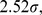 and a number like 50,000 links deviates by more than 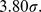 If the number of customer links is lognormally distributed, the cumulative probabilities corresponding to 5,000 and 50,000 links are 0.0058 and 0.000072, which is much lower than the probabilities that we actually observe, indicating that the number of customer links has a heavier upper tail than a lognormal distribution.

The CDFs of customer links show a linear relationship between the log of the number of links and the log of the corresponding cumulative probability for the number of links within the range of 80 to 50,000. The slope is around 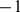 and is not significantly different from this value in each of the five years, that is,
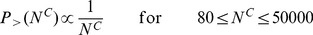
(3)where 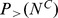 represents the probability that the number of customer links exceeds a certain value. Eq. (3) shows that 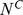 follows a power-law distribution and, more importantly, that its exponent is very close to unity. Power-law distributions with exponent 1 are found in various economic phenomena, including the distribution of city sizes, asset price changes, and firm sizes, a phenomenon referred to as Zipf's law. Most importantly, as shown by previous studies [23], firm sales follows Zipf's law, suggesting that the sales of a firm are related to the number of customers the firm has. We will come back to this issue in the later subsection headed “Implications for Firm Sales and Growth”.

Turning to the number of supplier links, we again find a linear relationship between the log of the number of supplier links and the log of the corresponding cumulative density, indicating that the number of supplier links also follows a power-law distribution. However, the slope of the linear relationship is much larger than that in the case of customer links, implying that the tail part of the supply link distribution is less fat than that of the customer link distribution. The slope associated with supplier linkages is about −1.5, so that the CDFs for the number of supplier links can be characterized by
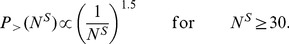
(4)Since the power-law exponent in this case exceeds unity, Zipf's law does not hold. Note that the power-law exponent 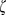 is related to the Gini coefficient, 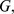 in the form 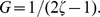 Therefore, the fact that the power-law exponent is larger for supplier linkages than for customer linkages implies that the Gini coefficient is smaller for supplier linkages and that, therefore, the number of supplier links across firms is less unequal than the number of customer links.

What explains this result? As emphasized in the recent literature on customer search models [24], [25], firms spend substantial resources on marketing to acquire as many customers as possible in order to increase their sales and profits. Such competition among firms produces winners with a large number of customers as well as losers with a small number of customers, resulting in huge inequality in the number of customers. In contrast, with regard to supplier linkages, firms have little incentive to increase their number of suppliers because it is not necessarily profitable to buy materials and intermediate products from more suppliers. It may even be the case that purchases from more suppliers increase the associated costs (e.g., shipping costs) and therefore reduce profits. Therefore, because firms do not compete to have as many suppliers as possible, the extent of inequality is not as high as that with regard to the number of customers.

#### How closely are firms interconnected?

**Figure 3:**
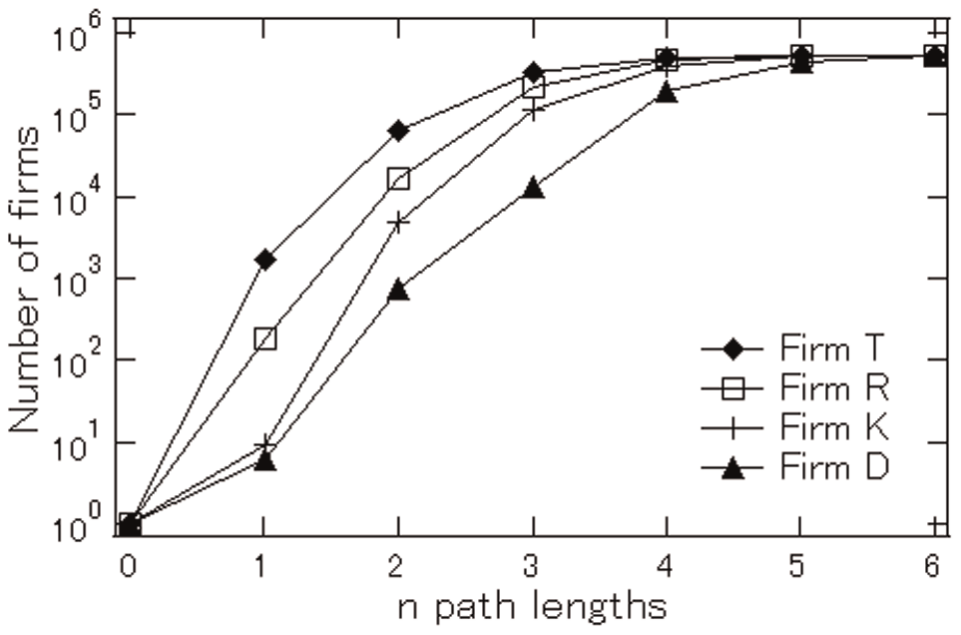
Number of firms connected to a particular firm by *n* path lengths. Firms *T*, *R*, *K*, and *D* are randomly picked from the sample, which consists of all the firms on the augmented customer/supplier lists.

To investigate how closely firms are interconnected, we use the augmented customer/supplier lists of partners mentioned in the previous subsection headed “Data” for the set of firms whose identification codes are listed in the customer and/or supplier lists of the other firms. The number of firms that appear in the augmented lists is 552,145 for 2008, 541,816 for 2009, 518,565 for 2010, 520,087 for 2011, and 525,836 for 2012. Specifically, we randomly pick four firms (Firms 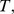 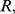 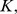 and 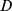) to examine the number of firms connected to a particular firm by one, two, three, or more path lengths. The result is shown in Figure 3. Firm 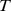 is connected to about 1,700 firms by one path length, but it is connected to more than 60,000 firms by two path lengths. The corresponding number for four path lengths increases to 503,796, which is only slightly less than the total number of listed firms. Thus, firm 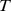 is connected to almost all the firms by four path lengths or less.

The fact that firm 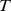 is connected to about 1,700 firms by one path length, which is much larger than the sample average presented in Table 1, suggests that it is extremely large. Given firm 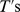 size and the fact that it is connected to about 1,700 firms by one path length, it may not be very surprising to find that it is connected to almost all the other firms by less than four path lengths. However, a more surprising case is Firm 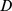, which is connected to only ten firms by one path length and, in fact, is very small with fewer than ten workers. Nevertheless, the number of firms to which Firm 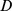 is connected is 746 for two path lengths, 13,519 for three path lengths, 196,799 for four path lengths, and 446,019 for five path lengths. Surprisingly, even a small firm like Firm 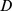 is connected to almost all the listed firms by five path lengths or less.

**Figure 4:**
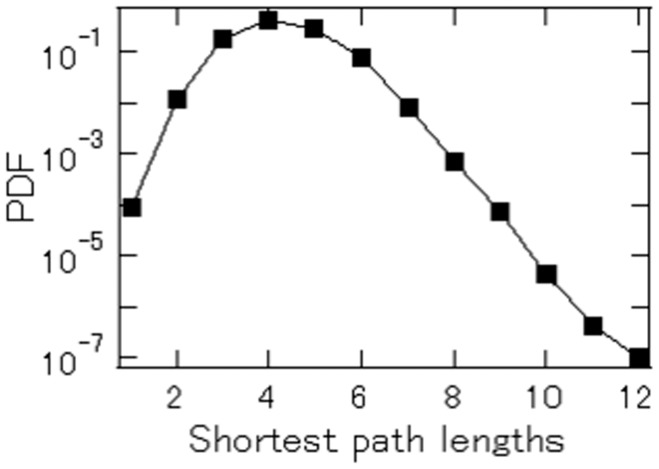
Distribution of the shortest path lengths for all pairs of firms. We pick firms that are on the augmented customer/supplier lists for each year in 2008–2011 *and* whose sales data are available for 1980–2009, the number of which is 134,067. We calculate the shortest path length for every pair of firms. There are 17.9 billion pairs.

We pick 134,067 firms that are on the customer/supplier lists for every year in 2008–2012, and then calculate the shortest path lengths for every pair of firms. We will focus on the same set of firms in the analysis in the later subsection headed “Implications for Firm Sales and Growth”. There are about 17.9 billion pairs and we find that 99.6% of all pairs are connected, but 0.4% cannot be connected regardless how long the path lengths are. Figure 4 shows the distribution of the shortest path lengths for those connected pairs. The mode of the distribution is four path lengths, and about 61.7% of the pairs are connected by four path lengths or less. Note that a similar feature a customer-supplier network is reported by [15] using a different dataset. Also note that previous studies including [16] apply the technique of community analysis to customer-supplier networks to find that path length tends to be shorter between firms belonging to the same industry or located in the same region.

Why are firms so closely interconnected? It is important to recall that the number of links, in the case of both customer and supplier linkages, follows a fat-tailed distribution. This indicates that there are some (although not many) firms with an extremely large number of links. The presence of such “hub” firms implies that even a small firm, like Firm 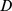 in Figure 3, is able to be connected to a large number of firms through these hub firms; that is, once a small firm finds a path reaching one of the hub firms (probably via several steps), it is then connected to the large number of firms to which the hub firm is linked. This kind of small-world phenomenon can be found for various economic and social networks [9], [10].

### The Evolution of Customer-Supplier Networks

A distinctive feature of our dataset is that it records information on linkages for five different years, allowing us to investigate not only the structure of customer-supplier networks at a particular point in time, but also their evolution over time. Some firms continue to buy from the same suppliers and sell to the same customers for a long period. However, other firms change their partners quite often. The duration of customer-supplier relationships influences how shocks are transmitted through the network. Suppose that a firm is hit by an adverse shock, and the firm reduces its production. If the relationships are all fixed and the network therefore is highly stable, then the shock to that firm spreads to downstream firms, which are also forced to reduce production. However, if relationships are flexible in the sense that firms can change their customers/suppliers easily (i.e., without incurring any large costs), downstream firms can easily establish new supplier links and thereby keep the shock from spreading.

To see how quickly customer andsupplier networks evolve over time, we present in Table 2 some statistics related to the turnover of customers and supplier links. Specifically, we identify customer links that appear on the augmented customer list of a firm in 2008 but not in 2009 and count them as link exits. Similarly, we identify customer links that do not appear in the augmented customer list of a firm in 2008 but do appear in 2009 and count them as link entries Links that appear in the firm's augmented customer list in both 2008 and 2009 are referred to as survivals. The table shows that the entry rate for 2008–2009 (the number of link entries in 2009 relative to the total number of links in 2008) is 10.8%, and the exit rate during the same period is 7.4%. Since the entry rate exceeds the exit rate, the number of links increases from 2008 to 2009 by 3.4%. On the other hand, the survival rate for 2008–2009 (i.e., the number of surviving links between 2008 and 2009 relative to the total number of links in 2008) is 92.6%, indicating that firms update their customer lists only partially within a year. Given that the survival rate falls to 87.2% for the two-year period from 2008 to 2010, 82.5% for the three-year period from 2008 to 2011, and 78.2% for the four-year period from 2008 to 2012, the survival rate for the next 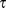 years, which we denote by 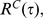 is estimated as
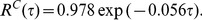
(5)Given the above relationship, simple calculation indicates that about 45% of links disappear over a decade and 70% over two decades. For supplier links, the entry and exit rates for 2008–2009 are 8.9% and 6.7%, respectively, and the survival rate is 93.3%, indicating a slightly lower turnover than for customer links. The survival rate for the next 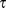 years, 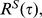 is given by
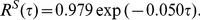
(6)


**Table 2:** Turnover of customer and supplier links.

Customer Links	Number of Links in the Initial Year	Net Increase	Entries	Survivals	Exits
Between 2008 and 2009	867,612	29,583	93,540	803,655	63,957
		(0.034)	(0.108)	(0.926)	(0.074)
Between 2008 and 2010	829,014	52,511	158,564	722,961	106,053
		(0.063)	(0.191)	(0.872)	(0.128)
Between 2008 and 2011	801,508	70,835	210,754	661,589	139,919
		(0.088)	(0.263)	(0.825)	(0.175)
Between 2008 and 2012	781,578	78,281	248,723	611,136	170,442
		(0.100)	(0.318)	(0.782)	(0.218)

Supplier Links	Number of Links in the Initial Year	Net Increase	Entries	Survivals	Exits
Between 2008 and 2009	864,814	19,416	77,154	807,076	57,738
		(0.022)	(0.089)	(0.933)	(0.067)
Between 2008 and 2010	830,486	32,667	128,810	734,343	96,143
		(0.039)	(0.155)	(0.884)	(0.116)
Between 2008 and 2011	801,210	46,418	173,515	674,113	127,097
		(0.058)	(0.217)	(0.841)	(0.159)
Between 2008 and 2012	779,470	56,970	210,670	625,770	153,700
		(0.073)	(0.270)	(0.803)	(0.197)

Note: The figures in parentheses show the ratio to the number of links in the initial year.

**Figure 5:**
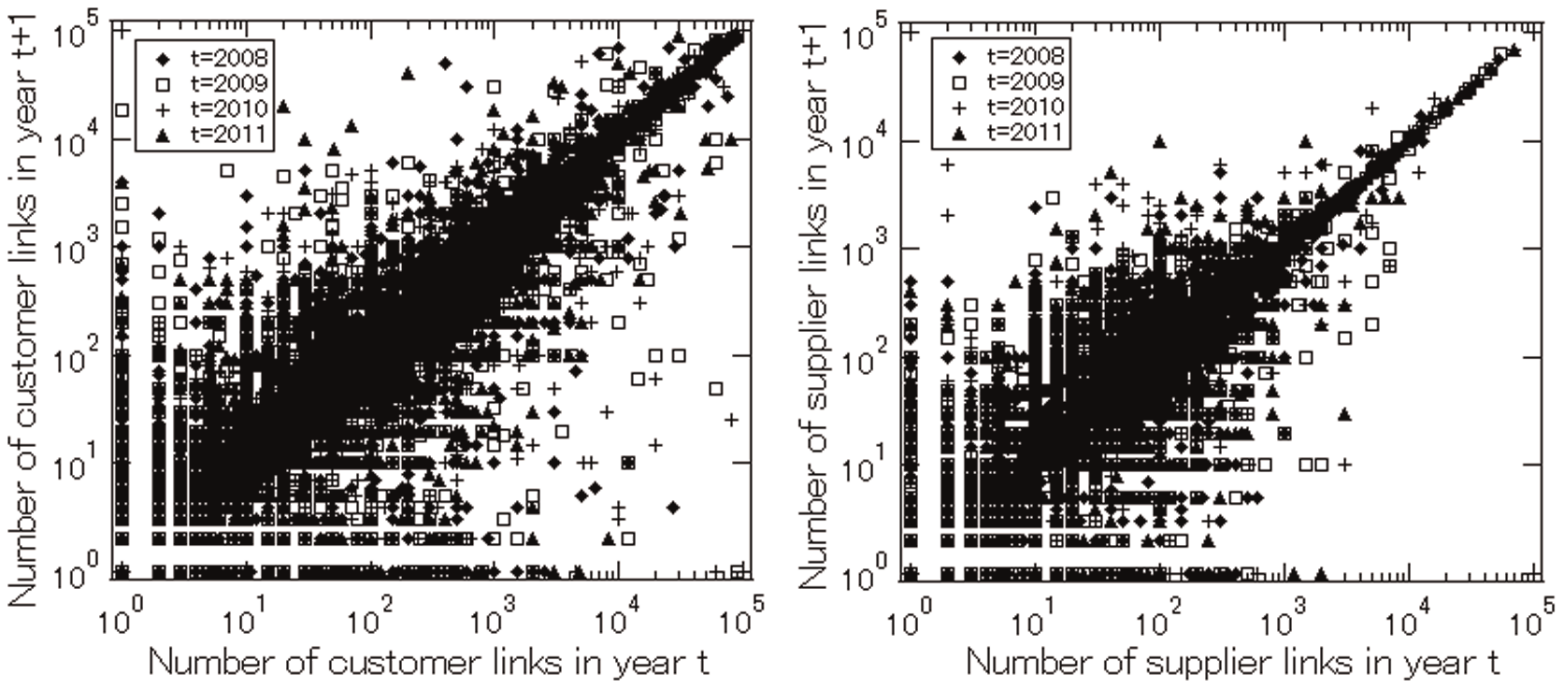
The number of links in year *t* on the horizontal axis versus the number of links in year *t+1* on the vertical axis. The upper and lower panels are for customer links and for supplier links respectively. The figures are produced using the *total* number of links, i.e., 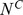 and 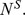 The number of firms used in the figures is shown in Table 2.

Next, we examine changes in the total number of links, i.e., 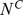 and 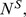 over time. We saw in Figure 2 that the distribution of the total number of links, for both customer and supplier linkages, does not change much over the five years. However, this does not necessarily imply that the number of links for each firm does not change much. For example, suppose a firm increases its links from 20 in year 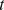 to 100 in year 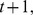 and another firm reduces its links from 100 to 20. In this case, the link distribution does not change at all between years 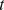 and 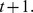 To see whether underneath the stable distribution there are changes in firm links that more or less offset each other, Figure 5 presents scatter plots for customer and supplier linkages respectively, showing the number of a firm's links in year 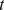 on the horizontal axis and the number of its links in year 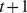 on the vertical axis. We see that the dots are concentrated on the 45 degree line for both customer and supplier links, indicating that for most firms the number of links remained unchanged. At the same time, there are also dots away from the 45 degree line; for example, for some firms, links increase by a factor of ten or even 100, while for others they decrease by similar factors to one-tenth or one-hundredth. Comparing the scatter plots for customer and supplier links, more dots are away from the 45 degree line for customer links, indicating that links with customers are more volatile than those with suppliers.

**Figure 6:**
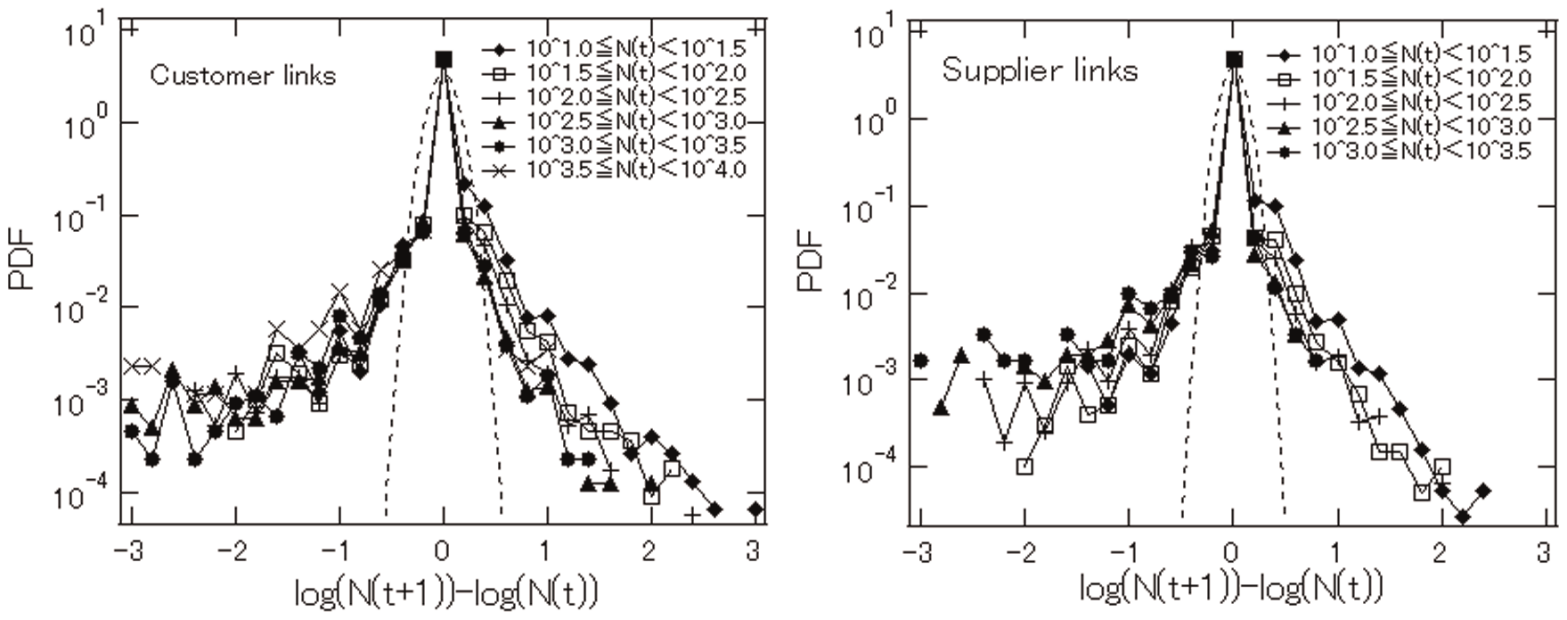
Distributions of link growth rates from year 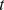 to year 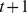 for customer links (upper panel) and for supplier links (lower panel). The dotted curve in the upper panel represents a normal distribution with a standard deviation of 0.12, which is the standard deviation estimated for the growth rate of customer links, while the dotted curve in the lower panel represents a normal distribution with a standard deviation of 0.10, which is the standard deviation estimated for the growth rate of supplier links.

To examine in more detail how firms' number of links changes over time, we show in Figure 6 the distributions of the annual growth rates for the number of customer links, 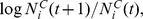 and for the number of supplier links, 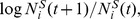 with the growth rates on the horizontal axis and the corresponding densities on the vertical axis. Note that there are eleven distributions in total in the two panels, each corresponding to a group of firms with a certain number of links in year 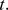 For example, the distribution labeled 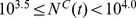 represents the distribution of the growth rates of the number of customer links from year 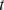 to year 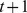 for firms with a number of customer links within the indicated range.

**Figure 7:**
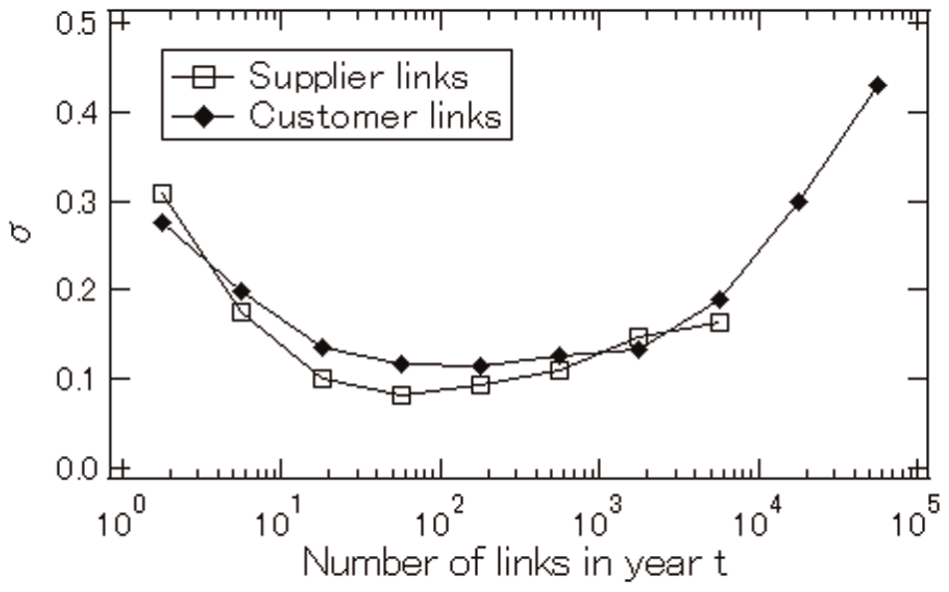
Relationship between the number of links in year 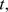 which is shown on the horizontal axis, and the standard deviation of link growth rates from year 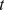 to year 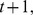 which is shown on the vertical axis.

Figure 6 shows the following. First, there is a clear peak in the distribution at densities corresponding to a growth rate of zero. The ratio of firms with a zero growth rate is 93.0% for customer links and 95.2% for supplier links. Second, each distribution has a fat upper tail. This can be seen more clearly if we compare the distributions with the dotted line representing a normal distribution with the same mean and standard deviation as observed in the data. Interestingly, the upper tail is even fatter for distributions of customer links than supplier links, suggesting again that fierce competition among firms to acquire new customers yields winners with very high growth in customer links as well as losers with very large negative growth. Third, the distributions do not depend much on the number of links in year 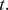 To show this more clearly, Figure 7 plots the number of links in year 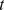 against the standard deviation of the growth rates of links from 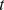 to 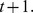 The figure shows that although the standard deviation is relatively high when the number of links in year 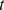 is either very small (i.e., below 10) or very large (above 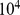), it is comparatively small and almost uniform for intermediate values.

To see what this almost uniform standard deviation means, let us consider a simple Poisson type situation. We assume that the number of attempts that a firm makes to acquire new customers in 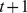 is proportional to the number of customers the firm has in 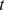. We denote the number of attempts by 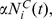 where 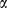 is a positive parameter. We also assume that the probability of success for each attempt is 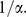 In this simple setting, the growth rate from 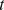 to 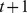 of the number of customers for a firm is, on average, unity, which is consistent with the empirical result shown in Figure 6. However, the standard deviation of the link growth rates is 
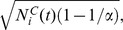
 indicating that the standard deviation is not invariant but decreases with 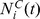 due to the law of large numbers, which is clearly inconsistent with the empirical result presented in Figure 7. The result shown in Figure 7 suggests that the law of large numbers does *not* hold in the data, so that the risk of losing many customers from 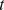 to 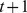 is not small even for firms with a large customer base in period 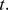


### Implications for Firm Sales and Growth

#### The relationship between customer links and firm sales

The sales of a firm in a particular year can be decomposed into two parts: sales to other firms as intermediate output (“intermediate demand” in the terminology of input-output analysis) and sales to non-firm sectors, including consumers, the public sector, and foreign buyers (“final demand”). The intermediate demand component of a firm's sales can be further decomposed into two determinants: the number of customer links and the average size of customer links (in terms of sales). In the terminology of economics, the number of customer links is the extensive margin, while the average size of customer links is the intensive margin. An important question to be asked is which of the two margins accounts for differences in the intermediate demand component of firm sales. In the context of international trade, this issue has been addressed by a number of studies including [26], [27], some of which show the relative importance of the extensive margin [27]. In the context of firm dynamics, some studies argue that the number of customer links plays a dominant role in explaining differences in firm sales [14], while certain anecdotal evidence suggests that having links of a larger size, which may reflect closer and longer-lasting ties with a particular partner, makes it possible for firms to achieve higher sales.

**Figure 8:**
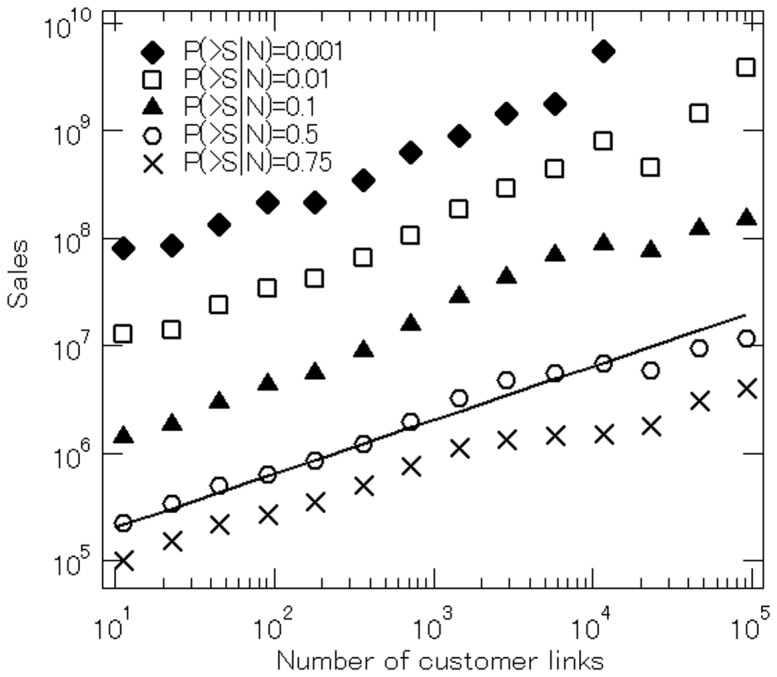
Firm sales conditional on the number of customer links. We define 14 bins of the same size in logarithm for the number of customer links and show various percentiles of the sales distribution for firms belonging to each bin, namely the 25th (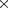), 50th (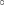), 90th (▴), 99th (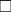), and 99.9th (♦) percentiles. The solid straight line is a reference line with a slope of 1/2.

However, to the best of our knowledge, researchers have access neither to information that makes it possible to decompose firm sales into final and intermediate demand nor to information on the size of customer links. Our dataset does not contain that kind of information either. However, we are still able to investigate how the number of customers for a firm is related to the sales of the firm. To this end, Figure 8 shows the relationship between the two, depicting the number of a firm's customers on the horizontal axis and the firm's sales on the vertical axis. More specifically, we define 14 bins of the same size in logarithm for the number of customer links and show various percentiles of the sales distribution for firms belonging to each bin, namely the 25th (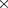), 50th (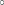), 90th (▴), 99th (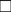), and 99.9th (♦) percentiles. As can be clearly seen in the figure, sales are positively correlated with the number of customer links. Moreover, a simple regression indicates that the median of the sales distribution in logarithm, denoted by 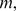 depends on the number of customer links. Specifically, the relationship can be expressed as follows:
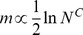
(7)Note that a similar linear relationship holds for the other percentiles, especially for the upper tail part, which is consistent with the results reported in the previous studies including [14], [29]. Eq. (7) implies that the variance in the log of sales is related to about 25 percent of the variance in the log of the number of customer links, suggesting that the extensive margin is relatively important. At the same time, however, Eq. (7) also indicates that a 10 percent increase in the number of customer links raises firms' sales only by 5 percent, implying that other determinants of firms' sales that are not controlled for in the regression may be inversely correlated with the number of customer links. For example, the size of customer links may be negatively correlated with the number of customer links; that is, firms with a larger customer base may have customer links of a smaller size. Alternatively, firms with a larger customer base may be located more upstream in customer-suppliers chains, so that they may have less opportunity to sell their products to consumers, etc., as final output.

#### Can customer-supplier links predict firm growth correlations?

Close interconnectedness among firms implies that an idiosyncratic shock to a firm could diffuse widely to other downstream firms through customer-supplier chains and, ultimately, result in fluctuations in the economy as a whole. As clearly demonstrated by the recent earthquake in Japan, the production activities of firms are closely correlated when these firms are “neighbors” in a customer-supplier network.

**Figure 9:**
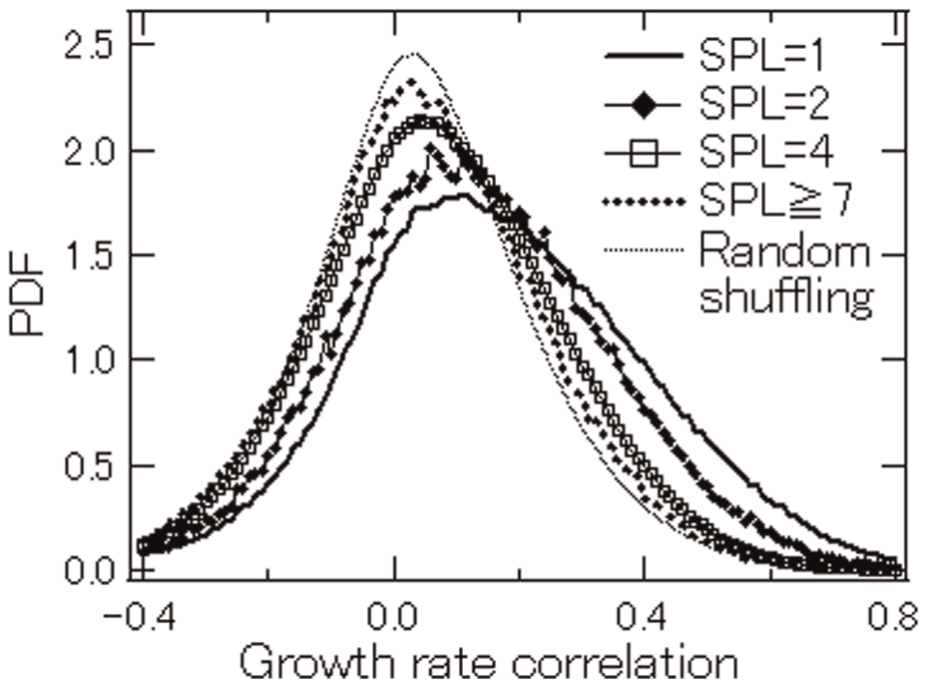
Distributions of growth rate correlations between two firms with different shortest path lengths. The thin dotted line labeled “random shuffling” represents the distribution for the case of random shuffling in which (1) we randomly pick two years for a particular firm, swap the growth rates for the two years, and repeat this for other pairs of years; (2) we conduct the same random shuffling for other firms until we have completely eliminated any correlation between the growth rates for any pair of firms.

To investigate such correlation in production activities in more detail, we compute the correlation in annual sales growth between two firms, firms 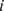 and 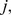 which is represented by 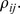 We do so for every year for all firms on the augmented customer/suppliers lists in 2008-2012 whose sales data are available for 1980–2009. (The number of firms that meet these criteria is 134,067.) We then examine how 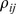 is related to the shortest path length between firms 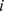 and 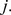 The results are shown in Figure 9, which depicts the distribution of 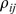 for firms that are connected by one path length (labeled “
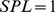
”), by two path lengths (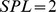), by four path lengths (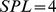), and by seven or more path lengths (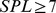). We find that 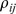 is distributed around zero in the case of 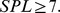 Here, we introduce random shuffling and eliminate growth rate correlations among firms as follows. For a particular firm, we randomly pick two years, swap the growth rates for the two years, and repeat this for other pairs of years. We do the same for all other firms until we have completely eliminated any correlation between the growth rates for any pair of firms. The distribution in the case of 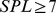 is almost identical to the distribution obtained by eliminating any correlations between firm growth rates by random shuffling, which is shown by the thin dotted line, indicating that there is no statistically significant correlation between the growth rates for firms 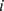 and 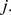 However, the distribution of 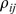 moves to the right in the case of 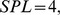 more to the right in the case of 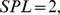 and even more to the right in the case of 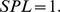 These results indicate that there is a positive and statistically significant correlation between the growth rates for firms 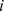 and 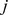 if they are close to each other in a customer-supplier network. Simple regression shows that the growth rate correlation between firms 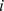 and 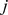 is related to the shortest path length between them as follows:
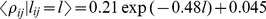
(8)where 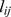 is the shortest path length between firms 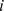 and 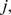 and 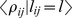 is the average of 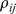 conditional on that the shortest path length between them is 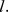 The first term in Eq. (8), 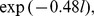 indicates that the growth rate correlation decreases with 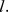


The positive constant term in Eq. (8), 0.045, indicates that the growth rates of firms 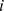 and 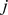 are positively correlated even when 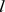 is very large, implying that part of the growth rate correlations may be due to factors that have nothing to do with customer-supplier chains. In fact, the growth rate correlation for pairs of firms which are not connected at all in the network (i.e., 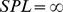) is, on average, 0.056, which is close to the constant term in Eq. (8). To examine the relationship between the growth correlation and the shortest path length in more details, we follow the recent literature on supply chains [17], [ 28] and assume that
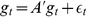
(9)where 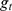 is a vector of firm growth rates 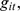 where 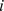 stands for the firm, so that 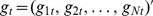), and 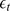 is a row vector representing shocks not stemming from customer-supplier chains (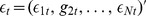). 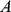 is an 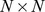 input-output matrix with typical element 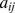 equal to 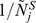 if firm 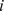 is the supplier of firm 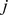 and zero otherwise. In the standard notation adopted in input-output analysis, 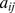 represents the share of input 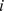 (i.e., commodity produced by firm 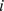) in the total intermediate input use of firm 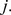 We have information on whether firm 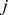 purchases something from firm 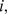 but no information on the amount of input 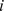 used in production of output 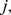 namely how thick each of the supply links is. We assume that the supply links of firm 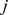 are of the same thickness, so that 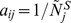 if firm 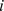 is the supplier of firm 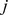 and zero otherwise. We assume further that 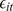 can be decomposed into shocks common to all firms, such as changes in monetary and fiscal policies, and idiosyncratic shocks:
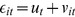
(10)where 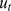 and 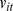 represent common and idiosyncratic shocks respectively, and 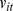 and 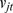 are uncorrelated. Using Eqs. (9) and (10), we decompose growth correlations into two parts: the correlation stemming from customer-supplier linkages, and the correlation due to common shocks. We first use 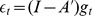 to recover 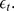 Then, we eliminate the simultaneous pairwise correlation between 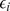 and 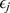 by randomly exchanging 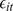 and 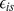 until the correlation is removed completely. We denote the uncorrelated new disturbance vector by 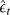 and define the new growth rate vector 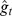 as 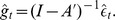 Note that in 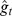 the growth rates for 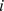 and 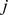 cannot be correlated through common shocks but may be correlated through customer-supplier linkages.

**Figure 10:**
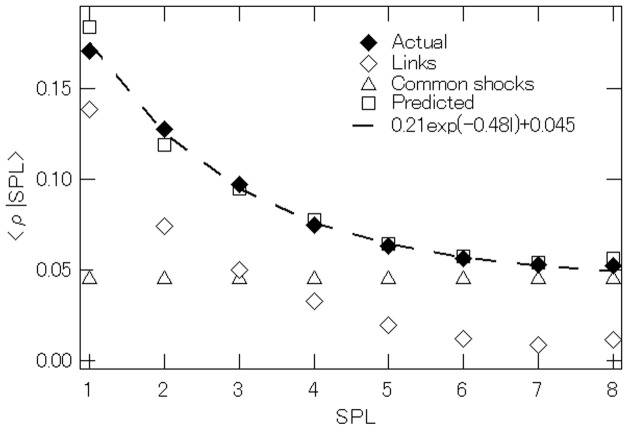
Average of the growth rate correlations between pairs of firms conditional on the shortest path length between the pairs. The figure shows the correlations obtained from the data (♦), the correlations through common shocks (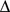), the correlations through customer-supplier links (◊), and the correlations through the sum of the two (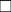).

The result of this exercise is presented in Figure 10, where the horizontal axis shows the shortest path length, while the vertical axis depicts the growth correlation conditional on the shortest path length. The result using actual growth rate data, 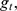 is represented by ♦ and shows that 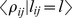 decreases with 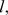 as we saw in Eq. (8). Next, the result for the growth rate correlations only through linkages, which are calculated using 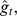 are shown by ◊. The result indicates that 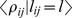 again falls with 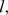 but this time it falls very close to zero when 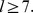 Finally, we add the estimate for the growth rate correlations through common shocks, 0.045 in Eq. (8), to the growth rate correlations through linkages. Doing so shows that the sum of the two, which is represented by 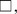 successfully generates the growth rate correlations observed in the data.

## Conclusion

In this study, we investigated the structure and evolution of customer-supplier networks in Japan using a unique dataset that contains information on customer and supplier links for more than 500,000 incorporated non-financial firms for the five years starting from 2008 to 2012. Our main findings can be summarized as follows. First, we show that the number of customer links is unequal across firms in the sense that the customer link distribution is substantially skewed to the right. The upper tail of the customer distribution is much thicker than that of a lognormal distribution and close to that of a power-law distribution with an exponent of unity (i.e., it follows Zipf's law). We interpret this as implying that competition among firms to acquire new customers yields winners with a large number of customers, as well as losers with fewer customers. We also show that the distribution for the number of suppliers across firms has a power-law tail, but the associated exponent is greater than unity, indicating that the number of supplier links is less unequal than the number of customer links. Second, we find that firms are closely interconnected with each other. Specifically, the shortest path length for any pair of firms is, on average, 4.3 links. Third, we show that in our observation period the frequency of link switching is limited and that, consequently, customer-supplier networks are fairly stable over time. Our estimates indicate that the survival rate for customer links (i.e., the rate at which existing customer links survive one more year) is 92 percent, while that for supplier links is 93 percent. Fourth, we find that the growth rates of a pair of firms tend to be more highly correlated when the two firms are closer to each other in a customer-supplier network (i.e., the shortest path length between the two firms is shorter), suggesting that a non-negligible portion of fluctuations in firm growth stems from the propagation of microeconomic shocks - that is, shocks affecting only a particular firm – through customer-supplier chains.

In this paper, we have focused on interfirm connections through customer-supplier relationships, but some previous studies, including [30]–[32], identify interfirm connections through stock price correlations. Specifically, these studies construct an interfrim network based on stock price correlations to show that firms with customer-supplier relationships, such as an automobile maker and a tire manufacturer which provides tires to the auto maker, tend to be close to each other (i.e., path length is short) even in the network constructed based on stock price correlations. However, these two interfirm networks are not necessarily closely related to each other. For example, it is known that, during stock price bubbles, stock prices of two firms were not correlated as suggested by customer-supplier links between them. Much remains to be done regarding how these two interfirm networks are related to each other.
